# Mitochondrial damage and “plugging” of transport selectively in myelinated, small-diameter axons are major early events in peripheral neuroinflammation

**DOI:** 10.1186/s12974-018-1094-8

**Published:** 2018-02-27

**Authors:** Marija Sajic, Keila Kazue Ida, Ryan Canning, Norman A. Gregson, Michael R Duchen, Kenneth J Smith

**Affiliations:** 1 Department of Neuroinflammation, Institute of Neurology (Queen Square), University College London, 1 Wakefield Street, London, WC1N 1PJ UK; 2 Cell and Developmental Biology, University College London, Gower Street, London, WC1E 6BT UK

**Keywords:** Experimental autoimmune neuritis (EAN), Phototoxic damage, In vivo, Confocal imaging, Mitochondrial, Small axons, Pain, Neuropathy

## Abstract

**Background:**

Small-diameter, myelinated axons are selectively susceptible to dysfunction in several inflammatory PNS and CNS diseases, resulting in pain and degeneration, but the mechanism is not known.

**Methods:**

We used in vivo confocal microscopy to compare the effects of inflammation in experimental autoimmune neuritis (EAN), a model of Guillain-Barré syndrome (GBS), on mitochondrial function and transport in large- and small-diameter axons. We have compared mitochondrial function and transport in vivo in (i) healthy axons, (ii) axons affected by experimental autoimmune neuritis, and (iii) axons in which mitochondria were focally damaged by laser induced photo-toxicity.

**Results:**

Mitochondria affected by inflammation or laser damage became depolarized, fragmented, and immobile. Importantly, the loss of functional mitochondria was accompanied by an increase in the number of mitochondria transported towards, and into, the damaged area, perhaps compensating for loss of ATP and allowing buffering of the likely excessive Ca^2+^ concentration. In large-diameter axons, healthy mitochondria were found to move into the damaged area bypassing the dysfunctional mitochondria, re-populating the damaged segment of the axon. However, in small-diameter axons, the depolarized mitochondria appeared to “plug” the axon, obstructing, sometimes completely, the incoming (mainly anterograde) transport of mitochondria. Over time (~ 2 h), the transported, functional mitochondria accumulated at the obstruction, and the distal part of the small-diameter axons became depleted of functional mitochondria.

**Conclusions:**

The data show that neuroinflammation, in common with photo-toxic damage, induces depolarization and fragmentation of axonal mitochondria, which remain immobile at the site of damage. The damaged, immobile mitochondria can “plug” myelinated, small-diameter axons so that successful mitochondrial transport is prevented, depleting the distal axon of functioning mitochondria. Our observations may explain the selective vulnerability of small-diameter axons to dysfunction and degeneration in a number of neurodegenerative and neuroinflammatory disorders.

**Electronic supplementary material:**

The online version of this article (10.1186/s12974-018-1094-8) contains supplementary material, which is available to authorized users.

## Background

Guillain-Barré syndrome (GBS) is a life-threatening, typically post-infectious, immune-mediated disease of the peripheral nervous system. Severe ascending muscle weakness, caused by dysfunction of peripheral motor fibers, is the best recognized and the most dramatic sign of the disease. Although most patients survive, about half retain a residual neurological deficit, which can be severe [1]. Sensory symptoms have been under-emphasized [2], but recent studies reveal that these are clinically relevant [3–5] and prevalent, with paresthesia being reported by 75% [2] of patients and pain by 89% [6]. Interestingly, these symptoms often precede muscle weakness. The presence of pain, paresthesia, and abnormal thresholds for thermal and mechanical stimuli [7] can occur several weeks prior to muscle weakness [8, 9]. Furthermore, following recovery, sensory impairment remains a long-term consequence of GBS, substantially impairing the quality of life [1, 10, 11]. Indeed, a prospective multi-center study conducted 2 years after disease onset identified sensory impairment in over half of the patient population [11], while another study reported that sensory deficits persist in the legs of 66% of patients between 3 and 6 years after the onset of GBS [12].

The early presence of sensory signs and symptoms and the long-term deficit suggest that small-caliber myelinated (Aδ) and unmyelinated axons (C fibers) are affected early in the pathogenesis of GBS. Indeed, the temporal pattern of functional impairment of these axons suggests that they are more vulnerable to inflammatory damage than the larger, motor axons. However, the pathophysiological mechanisms underlying the preferential vulnerability of small-diameter axons are unknown.

We have used confocal microscopy to compare the effects of experimental autoimmune neuritis (EAN), a model of GBS, on mitochondrial function and transport in myelinated, large-, and small-diameter axons in vivo at the time of onset of neurological deficits. We found that mitochondria depolarized, fragmented, and stopped trafficking early in the disease in all axons, but in small axons alone, the damaged mitochondria obstructed normal axonal transport of functional mitochondria, causing depletion of mitochondria distal to the site of damage. Mitochondrial blockage of myelinated, small-diameter axons may contribute to other disorders characterized by preferential degeneration of small fibers, including diabetic and chemotherapy-induced neuropathies.

## Results

### Inflammation at the onset of EAN reduces the number of transported mitochondria in peripheral nerve axons, but not their velocity

To investigate the mechanisms of inflammatory damage to peripheral nerve axons and axonal mitochondria, we immunized mito-S CFP^+^ mice with peripheral nerve myelin, and *Mycobacterium tuberculosis* and pertussis toxin as adjuvants. Such immunization results in an autoimmune response against proteins of peripheral nerve myelin in which spinal roots and peripheral nerves are infiltrated by inflammatory cells. EAN manifests first caudally, typically with temporary hind limb and tail weakness or paralysis, ascending rostrally, as occurs in GBS. To study the early pathological events in EAN, we imaged mitochondria in saphenous nerve axons at the first indication of clinical disease expression, typically weight loss and weakness of the tip of the tail. At this time, the vast majority of axons showed normal morphology, indistinguishable from that in time-matched asymptomatic controls and adjuvant controls. However, only in animals with EAN, we observed that the saphenous nerves contained a number of infiltrating cells with the characteristics typical of amoeboid macrophages, including the binding of isolectin B4 (IB4) (green fluorescent label). Also, time-lapse imaging revealed that these cells moved between individual fibers (Additional files 1 and 2: Videos S1 and S2). Interestingly, in some thin axons in the vicinity of the infiltrating cells, the distribution of mitochondria was uneven, with mitochondria in clumps instead of the typically uniform distribution of mitochondria seen in normal axons. Indeed, neither infiltrating cells nor clumps of mitochondria were found in control animals.

**Fig. 1:**
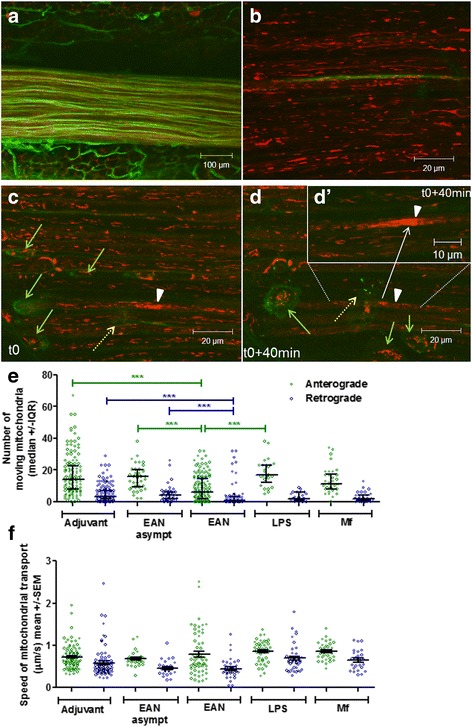
First signs of EAN are associated with inflammatory infiltration within the saphenous nerve. **a** Low-power image of dissected saphenous nerve, labeled in situ with TMRM (red) and IB4 (green), showing the outlines of nerve fibers and the connective tissues surrounding them. **b** High-power image of nerve fibers within the saphenous nerve in an adjuvant control animal, showing one IB4^+^ C fiber (green) and a number of elongated, polarized axonal mitochondria (red). **c** High-power image of nerve fibers within the saphenous nerve in a symptomatic animal on the first day of onset of EAN, showing a number of infiltrating inflammatory cells labeled with IB4^+^ (green arrows), within which mitochondria labeled with TMRM can be seen. In one axon (white arrowhead), polarized axonal mitochondria appear focally accumulated, as opposed to uniformly distributed mitochondrial in surrounding fibers. One of the inflammatory cells (dashed yellow arrow) can be seen juxtaposed to this focal accumulation (see also Additional file 1: Video S1, encircled). **d**, **d**′ After 40 min, the same inflammatory cell (dashed yellow arrow) is present at the same location (see also Additional file 2: Video S2). **d**′ The focal accumulation of axonal mitochondria shown from a different optical plane in order to visualize the mitochondria in the inset (see also Additional file 2: Video S2). This accumulation of mitochondria appears to have increased in comparison with the earlier time point (**c**). **e** In the symptomatic phase of EAN (*n* = 101 axons; 3 animals), the number of both anterogradely and retrogradely moving mitochondria in normal appearing axons was significantly decreased in comparison with adjuvant-only (*n* = 133 axons; 6 animals) and asymptomatic EAN animals (*n* = 44 axons; 3 animals) (***p < 0.001, two-way ANOVA). The systemic injection of LPS did not significantly change the number of transported mitochondria in comparison with asymptomatic EAN or adjuvant control animals and remained significantly higher than the number of anterogradely moving mitochondria in symptomatic EAN animals (***p < 0.001, two-way ANOVA). Similarly, incubation in vivo of saphenous nerve axons with macrophages activated ex vivo did not significantly change the number of moving mitochondria in comparison with controls, although the number of transported mitochondria in both directions in these animals tended to be lower than in controls and animals with systemic LPS. **f** The average speed of moving mitochondria was not affected by either of the studied conditions (*p* > 0.05, two-way ANOVA)

We wondered if the clumping of mitochondria may indicate a defect in mitochondrial transport, so we measured this in animals with EAN at disease onset, i.e., mild weakness of tail tip, comparing the findings with transport in immunized but asymptomatic animals (asymptomatic EAN), and in control animals that received adjuvant only (adjuvant controls). Control animals were examined on the same day as their cohort symptomatic animal or the next day. We found no differences between the number of transported mitochondria in adjuvant controls and asymptomatic EAN animals, in either the anterograde (towards the peripheral terminal) or the retrograde (towards the dorsal root ganglion (DRG)) directions. The median number of anterogradely transported mitochondria in adjuvant controls and asymptomatic mice with EAN was 14 (interquartile range (IQR) = 8–22) and 16 (IQR = 9–22), respectively, with the median number of retrogradely transported mitochondria in the two types of control equal to 3 (IQR = 2–7) and 4 (2–6) respectively. However, in animals expressing a neurological deficit, the median number of anterogradely transported mitochondria was reduced by more than half to 6 (2–14.5) in comparison with control groups (Fig. 1a, *p*< 0.001, non-parametric one-way ANOVA, Dunn’s post-test). The number of retrogradely transported mitochondria [(1 (0–3)) was also significantly lower than in control groups (*p* < 0.001, non-parametric one-way ANOVA, Dunn’s post-test). Therefore, at the onset of disease expression, the observations reveal infiltrating inflammatory cells within the perineurium and a significant reduction in axonal mitochondrial transport. Interestingly, the overall axonal structure in these fibers appeared normal, and the mitochondrial distribution was unaffected despite the reduction in axonal mitochondrial transport.

As the systemic inflammation alone (adjuvant controls) did not alter mitochondrial transport, we postulated that the infiltrating population of inflammatory cells may be responsible for the observed result. To determine whether the presence of inflammatory cells in the vicinity of axons may cause impairment of mitochondrial transport within those axons, we examined mitochondrial axonal transport in two different experimental paradigms. First, to exclude the possibility that systemic inflammation affects mitochondrial transport, we induced systemic inflammation by an intraperitoneal injection of lipopolysaccharide (LPS). Two to 4 h after LPS injection, when animals exhibit sickness behavior (i.e., reduced activity, piloerection, hyperventilation), we measured mitochondrial transport in saphenous nerve axons. The number of transported mitochondria was similar to that in adjuvant controls and asymptomatic EAN animals, and anterograde and retrograde transport was similar to controls (median anterograde and retrograde transport (±IQR) = 12 (5–17) and 2 (1–6) respectively (Fig. 1a). We then examined whether the presence of inflammatory cells infiltrated amongst the axons impaired mitochondrial transport, as seen at the onset of EAN. We mimicked inflammation in the peripheral nerve by incubating the saphenous nerve axons with activated macrophages. For this purpose, we isolated peritoneal macrophages from naïve mice and incubated them overnight with sciatic nerve excised from another C57BL6 mouse, in the presence of LPS. On the next day, we opened the perineurium of saphenous nerve axons in vivo and exposed them to 500,000 of these activated macrophages which were re-suspended in 200 μl of supernatant. We measured axonal transport 1 h after adding the macrophages to exposed axons. In comparison with adjuvant control and asymptomatic mice, this “applied infiltration” of saphenous nerve by activated macrophages reduced the number of mitochondria moving both anterogradely [median (IQR) = 11 (8–17.5)] and retrogradely [median (IQR) = 2 (1–4)], although this reduction did not reach statistical significance (Fig. 1e) in comparison with either adjuvant control or asymptomatic animals.

We investigated whether the decrease in the number of transported mitochondria was a result of a reduced speed of mitochondria, but we found no differences in transport speed in either direction between the groups (Fig. 1f).

### Inflammation during the symptomatic phase of EAN is associated with mitochondrial fragmentation, loss of mitochondrial membrane potential, and focal accumulations of damaged mitochondria

**Fig. 2:**
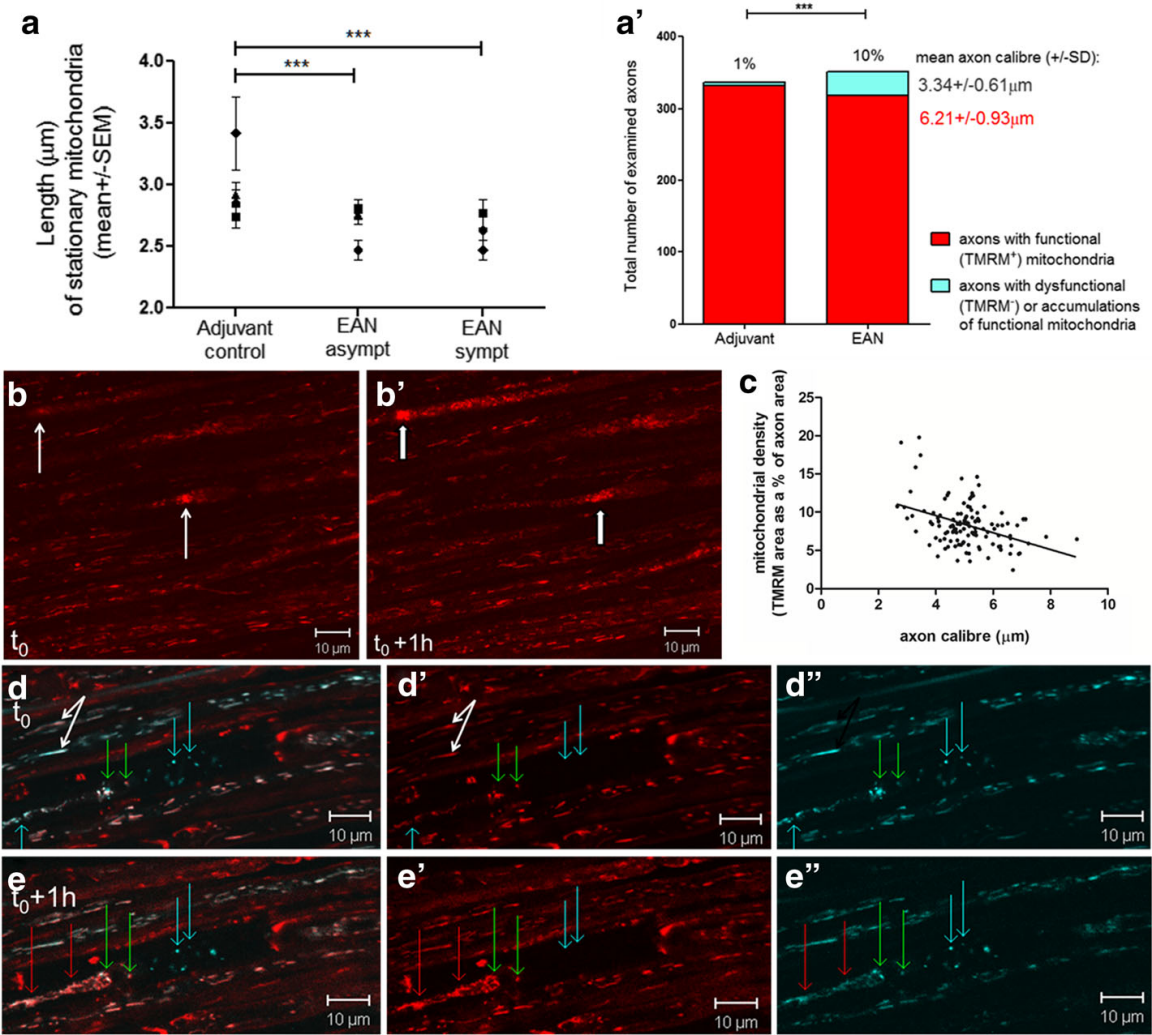
Inflammatory infiltration within peripheral nerve during EAN is associated with changes in mitochondrial distribution and axonal transport of mitochondria. **a** Polarized stationary mitochondria in axons of animals with either symptomatic or asymptomatic EAN are significantly shorter than in adjuvant controls (*p* < 0.0001, one-way ANOVA). **a**′ Focal accumulations of mitochondria are found primarily in small-diameter (mean ± SD = 3.34 ±  0.61 μm) axons; there was statistically significant difference between the total number of axons with detected accumulations found in adjuvant control (*n* = 6 animals) vs. EAN (*n* = 3 animals) groups (*p* < 0.001, Student’s *t* test. **b** The symptomatic phase of EAN is associated with focal accumulations of mitochondria (thin arrows), and these accumulate further over time (e.g., after 1 h, shown in **b**′, thick arrows), primarily by the addition of anterogradely trafficking healthy mitochondria, as observed by time-lapse imaging, in vivo. **c** In control axons, the density of polarized mitochondria tends to decrease with increasing caliber of axons (*p* < 0.0001, *r*^2^ = 0.16, Pearson correlation) indicating space restrictions for axonal mitochondrial transport in small-diameter axons. **d**–**e**″ Healthy axonal mitochondria are electrically polarized (i.e., they fluoresce red, TMRM^+^), appearing pale/white (white arrows) when they overlap with the blue fluorescence of CFP^+^ mitochondria. In animals with symptomatic EAN, some mitochondria become fragmented (short and round) but retain membrane potential (green arrows) whereas other mitochondria appear clearly damaged, not only appearing small and round, but also depolarized (TMRM^−^, blue arrows). Such mitochondria appear immobile, remaining stationary at the site of damage for at least 1 h (blue arrows). Healthy mitochondria join the accumulations (red arrows) but appear to become trapped at the site of damage

Examination of the length of axonal mitochondria revealed that stationary mitochondria at the onset of EAN were marginally shorter (2.6 ± 1.2 μm) than mitochondria in axons from animals with asymptomatic EAN (2.7 ± 1.3 μm; *p* < 0.01, one-way ANOVA, Dunns’ post-test), but significantly shorter than mitochondria in adjuvant controls (3 ± 1.5 μm, *p* < 0.001) (Fig. 2a). This finding indicates that immunization with peripheral nerve antigens may tilt the mitochondrial fusion/fission balance in favor of fission, even when signs of the disease are undetectable or barely detectable. As increased mitochondrial fission has been associated with mitochondrial dysfunction in neurodegenerative diseases [13–15], we next examined whether axonal mitochondria maintained their mitochondrial membrane potential (Fig. 2a–b′). Using transgenic mice that constitutively express cyan fluorescent protein in axonal mitochondria independently of mitochondrial membrane potential (i.e., both functional and dysfunctional axonal mitochondria appear blue), we applied the potentiometric dye TMRM (red) to the axons to distinguish functional (positive for CFP with accumulated TMRM, appearing pink or white) from dysfunctional mitochondria (positive for CFP but failing to accumulate TMRM, appearing blue) in vivo. In healthy controls and asymptomatic animals, the vast majority of axons were characterized by mitochondria of various lengths, amongst which both elongated and round shorter mitochondria were clearly labeled with both CFP and TMRM (Fig. 2d, white arrows). However, in animals with EAN, about 10% of axons showed an atypical, focal accumulation of mitochondria (Fig. 2b–e, arrows), and within these accumulations, depolarized mitochondria (blue) were stationary throughout the period of observation (typically over 2 h). Also, the depolarized mitochondria were fragmented, typical of damaged mitochondria [16]. Indeed, given that the fragmented and depolarized mitochondria do not participate in mitochondrial fusion, this effect of inflammation may have contributed to the perceived shortening of axonal mitochondria mentioned above. Interestingly, the focal accumulations, consisting of a mixture of depolarized and polarized mitochondria, appeared gradually to increase in size (Fig. 2b and b′). Furthermore, time-lapse imaging revealed that the accumulations occurred predominantly, although not exclusively, by anterogradely moving, polarized, mitochondria joining the “road block” (Additional files 3 and 4: Videos S3 and S4). Interestingly, only small-diameter myelinated axons seemed to be affected by mitochondrial accumulations (Fig. 2a′). We then measured the mitochondrial density in axons of different calibers and found that, indeed, small-caliber axons tend to host a greater density of mitochondria than large-caliber axons (Fig. 2c, *p*< 0.0001, *r*^2^ = 0.16). The combination of small-diameter and greater mitochondrial density makes small-diameter axons especially vulnerable to plugging when mitochondria are damaged and transport ceases.

### Focal mitochondrial damage increases anterograde mitochondrial transport into the damaged area but causes accumulations of mitochondria in small-diameter fibers

**Fig. 3:**
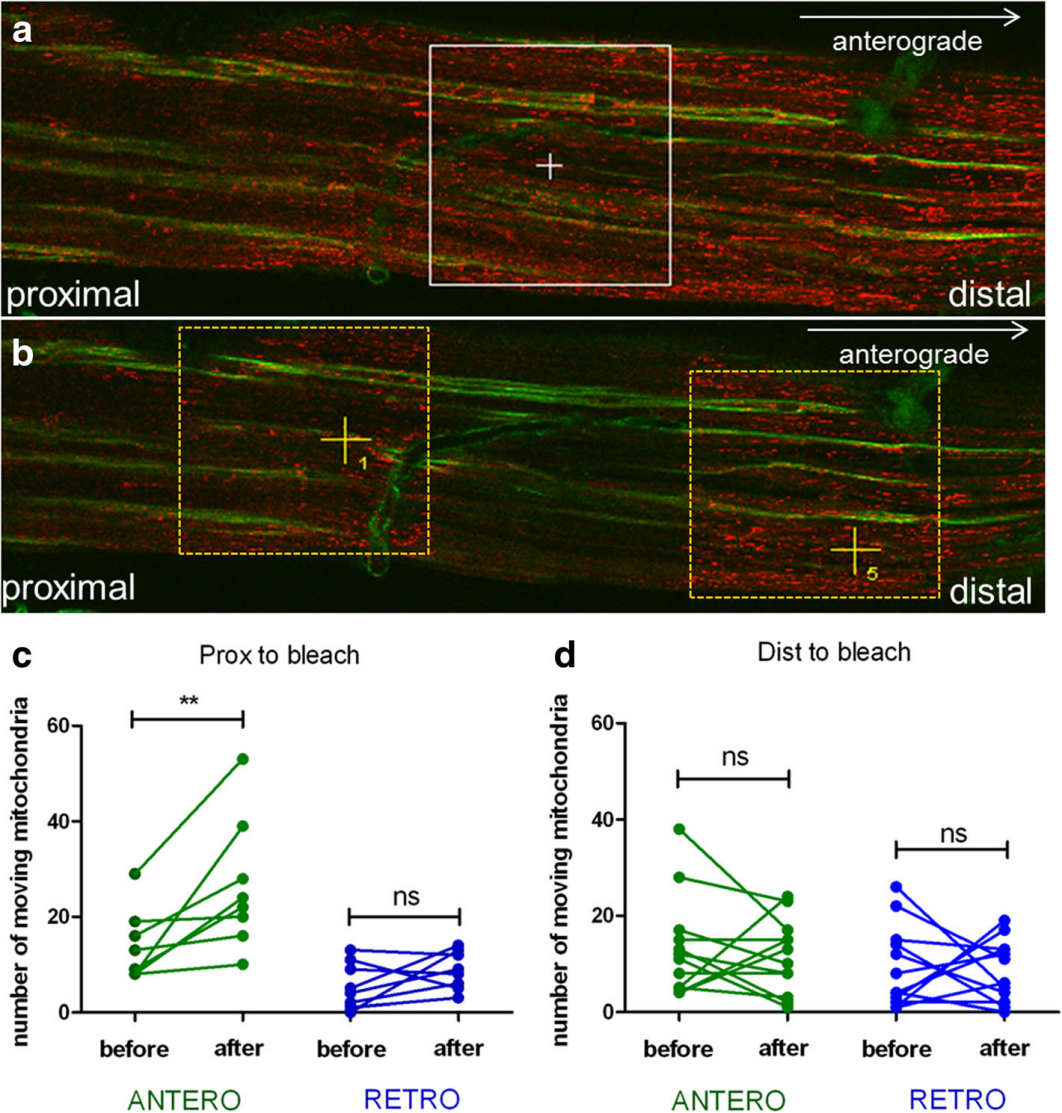
Depletion of functional mitochondria from the photo-bleached segment of axons induces a compensatory increase in anterograde mitochondrial transport proximal to the bleached site. **a** Tile image of saphenous nerve prior to photo-bleaching labeled with TMRM (red; healthy mitochondria) and IB4 (green; IB4^+^ C fibers, connective tissue); area exposed to the photo-bleaching is outlined in white. **b** Tile image of saphenous nerve immediately after the photo-bleaching, labeled with TMRM and IB4. Axons traversing the photo-bleached area and areas proximal (region 1 outlined in yellow) and distal (region 5 outlined in yellow) to the photo-bleached area were used for measurements of mitochondrial transport before and after the photo-bleach. **c** In axons proximal to the photo-bleached area, the number of transported mitochondria was measured before and after photo-bleach. The number of anterogradely moving mitochondria was significantly greater (**p = 0.008, repeated measures ANOVA; *n* = 8) after the photo-bleach than before the photo-bleach, whereas there were no differences in numbers of mitochondria moving retrogradely before and after the photo-bleach. **d** In axons distal to the photo-bleached area, there were no statistically significant differences in the numbers of mitochondria moving in direction either before or after the photo-bleach (*p* > 0.05, repeated measures ANOVA, *n* = 12)

We postulated that damaged mitochondria might cause a road block in the path of incoming transported axonal mitochondria. To test this hypothesis, we induced localized mitochondrial damage by photo-toxic bleaching (561 nm) in a well-defined area (Fig. 3, 124 × 124 μm region in A), and measured mitochondrial transport distally and proximally to the bleached region, before and shortly after damage (Fig. 3, regions 1 and 5 in B). We found that distal to the site of photo-toxic mitochondrial damage, the number of anterogradely and retrogradely moving mitochondria did not significantly change (Fig. 3d). However, proximal to the damage, there was a clear increase in anterograde transport in all examined axons (Fig. 3c, *p* = 0.008, repeated measures ANOVA). Thus, the damaged region of the axons seems repopulated mainly by the increase in number of anterogradely trafficking mitochondria moving into the region. The preponderance of anterogradely moving mitochondria into the photo-bleached region can be observed in time-lapse images of the region obtained within a few minutes after the photo-toxic depletion of mitochondria (Additional file 5: Video S5).

**Fig. 4:**
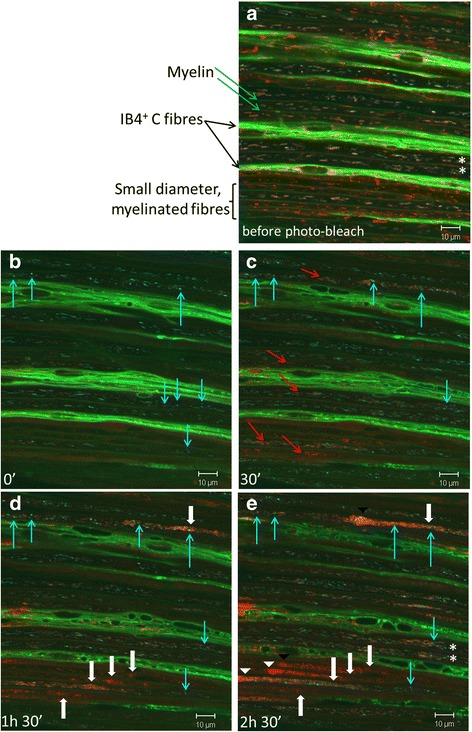
Mitochondria damaged by photo-bleaching remain at the site of damage obstructing the passage of cargoes along the small-diameter axons, resulting in a selective block of transport in small-diameter fibers. Before the photo-bleaching (**a**), axonal mitochondria appear healthy or electrically polarized (i.e., they fluoresce red, TMRM^+^), appearing pale/white when they overlap with the blue fluorescence of CFP^+^ mitochondria. Photo-bleaching induces fragmentation and damage of mitochondria (blue arrows, **b**–**d**) which remain at the site of damage for over 2 h (**e**). Depletion of functional mitochondria from the photo-bleached segment of axons induces a compensatory increase in anterograde mitochondrial transport proximal to the bleached site, resulting in re-population of these segments with healthy (TMRM^+^; red arrows, **c**–**e**) mitochondria. This mechanism successfully replenishes the mitochondria in axons of large and medium diameter (white asterisks, **a**, **e**). However, in small axons, the damaged mitochondria appear to block the passage of healthy mitochondria, resulting in proximal mitochondrial accumulations (white arrows in **d**, white arrowheads in **e**), and paucity of healthy mitochondria distal to the transport block—note the lack of TMRM^+^ mitochondria distal to white arrows, while CFP^+^ mitochondria remain stationary. Average diameter (±SD) of the fibers with mitochondrial transport block 2.5 h after bleaching was 3.02 (± 0.7) μm (*n* = 18 axons from three separate experiments)

Within the region subjected to photo-bleaching, all the mitochondria initially appeared polarized and uniformly distributed along the axons (Fig. 4a), but then, as expected following photo-bleaching, they all promptly became depolarized (TMRM^−^, Fig. 4b). The majority of the mitochondria retained their original shape and size, but some mitochondrial fragmentation occurred, including the formation of a small number of short, round mitochondria typical of the damaged form [16] (Fig. 4, blue arrows in B). Over the following hours, the vast majority of mitochondria within the photo-bleached area underwent fragmentation. These CFP^+^ TMRM^−^ mitochondrial fragments remained stationary for at least 2.5 h (Fig. 4, blue arrows in C, D, and E). As mentioned above, the photo-bleaching induced an increase in anterograde mitochondrial transport, and these polarized, anterogradely transported mitochondria began to repopulate the damaged region. However, the passage of the healthy, polarized mitochondria towards the distal parts of the axons was hindered by the stationary fragments of mitochondria generated by the photo-bleaching, resulting in the formation of focal mitochondrial accumulations consisting of both polarized and depolarized mitochondria (Fig. 4, white arrows in D). These accumulations steadily increased in size, mainly growing in the proximal direction (Fig. 4, white arrowheads in E), while the distal parts of the axons became depleted of mitochondria. Interestingly, accumulations of mitochondria only occurred in small-diameter, myelinated, fibers measuring 3.02 (± 0.7) μm (mean ± SD, *n* = 18 axons from three separate experiments). In larger axons, measuring 4.24 (± 1.15) μm (mean ± SD, *n* = 35 axons from three separate experiments), although some healthy, polarized mitochondria were blocked by damaged fragments, the majority by-passed these “road blocks” to travel onward to the distal axon (annotated by asterisks in Fig. 4). There was a statistically significant difference between the calibers of the axons that were differentially affected by laser photo-toxicity (*p* < 0.001, Student’s *t* test).

## Discussion

Clinical and pathological studies implicate dysfunction of small-diameter fibers in the early neurological signs of the most common form of acute peripheral inflammatory polyradiculoneuropathy, Guillain-Barré syndrome (GBS). In this study, we reveal how inflammation within the saphenous nerve is associated with mitochondrial dysfunction in small-diameter fibers, in vivo. The effects involve a sequential process including (i) loss of mitochondrial membrane potential followed by mitochondrial fragmentation, (ii) accumulation of mitochondrial debris at the site of damage, (iii) compensatory increase in anterograde mitochondrial transport proximal to the site of damage, (iv) obstruction of incoming transported mitochondria by the debris, and (v) mitochondrial depletion distal to the site of initial damage. We show that the consequences of this sequence of events are different in small-diameter myelinated axons compared with medium- and large-diameter axons in that greater mitochondrial density and space restrictions render small-caliber axons becoming more susceptible to a complete block of axonal transport of mitochondria at the site of initial mitochondrial damage.

### Association between inflammation and mitochondrial damage

At the onset of the signs of EAN, we observed that some axonal mitochondria appeared fragmented and depolarized, and that these were always associated with immobile inflammatory cells that were closely juxtaposed to the axon concerned. It was not clear whether the inflammatory cells were attracted by signals from the damaged axon, or whether the mitochondrial damage was a consequence of the attachment of the inflammatory cell to the axon as a consequence of the anti-nerve immune response, but it seems reasonable to speculate that in this model of active immunization, it was the inflammatory cells that were the instigators of the damage. Indeed, we have not observed mitochondrial damage or disruption of mitochondrial transport in any of the control groups of animals, i.e., animals that received only adjuvants, pyrogenic doses of LPS i.p., or application of in vitro-activated macrophages. Early mitochondrial damage in the context of neuroinflammation has been documented in EAE [17, 18], as well as in MS [19, 20], where soluble inflammatory mediators and reactive oxygen species have been implicated.

### Consequences of focal mitochondrial damage for the axons

The likely consequences of loss of functional mitochondria to the affected axon are multiple and complex. One of the most obvious is an energy crisis, with resulting Ca^2+^ toxicity due to depletion of the ATP required for Ca^2+^ homeostasis and the release of Ca^2+^ from damaged mitochondria [21]. It is well established that both a lack of ATP and high Ca^2+^ concentration are incompatible with long-term axonal survival [22, 23]. Thus, unsurprisingly, an increase in ADP [24] and Ca^2+^ have both been implicated in regulating mitochondrial axonal transport towards the area of high energy demand [25]. Such regulated, directional transport of functional mitochondria is vital for axonal survival under different physiological [26] and pathological conditions [21, 27, 28]. In agreement with these observations, made in ex vivo models, is our finding that focal, photo-toxic damage of axonal mitochondria rapidly induces an increase in anterograde transport of functional mitochondria towards the site of damage, in vivo*.* The supply of these mitochondria to the affected area would provide the ATP necessary to handle excess Ca^2+^, preventing axonal dysfunction, and possibly degeneration.

However, another challenge for axons affected by focal mitochondrial damage is the effective disposal of mitochondrial debris. In vitro studies have indicated that damaged axonal mitochondria are transported retrogradely to the cell body in order to be degraded [29]. It was later observed, in cultured hippocampal neurons, that autophagosome formation and subsequent mitophagy may occur without additional transport of damaged mitochondria, i.e., in situ [30]. Indeed, our study shows that in long, adult axons in vivo, damaged mitochondria are not removed from the site of damage and transported elsewhere for at least 3 h following the initial damage. The advantage of the in situ degradation of mitochondria is rapid neuroprotection against oxidative damage without energetically expensive and lengthy retrograde axonal transport [31]. Indeed, the mechanism underlying the detachment of depolarized mitochondria from microtubules, thereby preventing further axonal transport, has been described in rat hippocampal cultures. Namely, depolarized mitochondria are targeted by the PINK1/Parkin pathway, which subsequently initiates proteasome-dependent degradation of the mitochondrial adaptor protein Miro, resulting in release of the motor protein kinesin from mitochondrial surface [32]. Once detached from kinesin, depolarized mitochondria become arrested and likely stabilized in situ by cytoskeletal neurofilament [33].

An important finding of our study is that the presence of mitochondrial debris, exacerbated by a compensatory increase in axonal transport of functional mitochondria, represents a challenge for axons, especially myelinated axons of small caliber. It should be noted that by small-caliber axons, we do not refer to the classical nociceptors, the unmyelinated (or C) fibers, although a proportion of these axons is strongly, specifically labeled with IB4. Indeed, we have used another characteristic of IB4 to faintly label myelin, in order to visualize and measure the caliber of the small-caliber myelinated axons. By using this method, the caliber of any myelinated axon can be precisely measured, for example using the ZEN imaging software. The increased influx of functional mitochondria mentioned above is likely designed to improve the bio-energetic profile of the affected area of the axon; its spatial co-localization with the stationary mitochondrial debris may be counterproductive. Namely, the accumulated mitochondrial debris in small-diameter axons seems physically to obstruct the passage of the incoming, functional mitochondria from reaching parts of the axon distal to the affected site. In a similar way, a car breakdown on a small road will obstruct passing cars causing a traffic jam, whereas the same car on a wide road will have little consequence. Similarly, in medium- and large-caliber axons, the functional mitochondria can bypass damaged mitochondria. Thus, it appears that the coincidence of damaged mitochondria and a compensatory increase in axonal transport of functional mitochondria represents a challenge axons for small caliber. Indeed, we have shown that mitochondrial density is greater in the small-caliber axons than in the larger ones, which exacerbates the problem.

Further to the accumulation of mitochondrial debris, the damage to mitochondria within the axons will activate mechanisms for removal of such debris, for example via activation of PINK1/Parkin pathway, as mentioned above. The consequence of activation of PINK1/Parkin pathway is the formation of autophagosomes. Our and other recent studies strongly suggest that such formations will form locally, and this will further increase the mass of heterogeneous material likely to obstruct transport in small-caliber axons. It is easy to envisage the formation of a vicious circle aggravating transport deficits following an inflammatory insult and reducing the possibility of recovery. This consideration highlights the opportunities that may arise from enhancing the removal of debris material, such as improved mitophagy and autophagy strategies, in neurodegenerative diseases. Indeed, the distal segments of the affected small-diameter axons had already become depleted of functional mitochondria upon first observation, and this problem got worse during our observation period. Therefore, in vivo, only a few hours may be a sufficient time period for the consequences of blocked axonal transport and metabolic disturbances to become exacerbated. The depletion of mitochondria in the distal axon is likely to contribute to the long-term dysfunction and distal degeneration resulting from inflammatory insult. Indeed, sensory impairment remains a long-term consequence of GBS [10, 11], and it correlates with the density of intra-epidermal nerve fibers (IENF) [34], suggesting that the dysfunction is progressive, finally resulting in degeneration of the distal axon.

It seems likely that mitochondrial damage and distal depletion of mitochondria precipitated by selective obstruction of small-diameter myelinated axons could play a part in a number of other inflammatory conditions characterized by small fiber degeneration. Indeed, it has been shown in cultured dorsal root ganglia expressing small fiber neuropathy-associated G856D mutant Nav1.7 channels that degeneration of neurites [35] is promoted by a decrease in ATP levels and increase in intracellular calcium levels [Ca^2+^]_i_, which would be expected as a consequence of mitochondrial damage and depletion. Selective loss of small-diameter myelinated axons occurs in CNS in multiple sclerosis [36], and it is especially prominent in small fiber neuropathies (SFN), which are often characterized by pain and autonomic symptoms, including diabetic neuropathy [37–39], chemotherapy-associated neuropathy [40], HIV [41], systemic lupus erythematosus [42], paraneoplastic syndrome [43], sarcoidosis [44], drug toxicity [45–47], hereditary neuropathies [48], familial amyloidosis [49], and inflammatory peripheral neuropathy and Guillain-Barré syndrome (GBS) [42]. The pervasive vulnerability of small-diameter axons is striking, and the mechanisms described in this paper may contribute to their selective vulnerability. If so, research into therapeutic strategies to overcome the formation of a mitochondrial plug, such as stimulating the degradation of mitochondrial debris, may be beneficial target for treating peripheral neuropathies.

## Conclusion

The inflammatory insult creates debris of stationary, damaged mitochondria within axons. In small-diameter myelinated axons, the combination of a space restriction and a high density of mitochondrial debris together result in plugging of axons with debris which in turn further obstructs axonal transport and hinders repopulation of the affected segments with healthy mitochondria. The long-term depletion of mitochondria from affected portion and distal axons may contribute to the metabolic impairment, long-term dysfunction, and distal axonal degeneration.

## Methods

### Animals

All animal experiments were carried out according to the 1986 Animals (Scientific Procedures) Act, UK, and were approved by the institutional ethics committee. Mice used in this study were male, 8–12 weeks old, and either Thy1-CFP-S-positive or Thy1-CFP-S-negative littermates (Jackson laboratories strain designation: B6;CB-Tg (Thy1-CFP/COX8A)S2Lich/J). In CFP-S mice, the cyan fluorescent protein is constitutively expressed only in axonal mitochondria, affecting approximately 40–60% of them, and thus, the label was used to distinguish axonal from Schwann (and other) cell mitochondria. As the CFP labeled mitochondria fluoresce blue regardless of their membrane potential, we used another probe tetramethylrhodamine methyl ester (TMRM, Invitrogen, UK) which partitions into polarized mitochondria to label polarized (probably healthy) mitochondria selectively.

### Induction and assessment of experimental autoimmune neuritis (EAN)

EAN was induced in 8–12-week-old Thy1-CFP-S-positive and Thy1-CFP-S-negative littermates (Jackson laboratories strain designation: B6;CB-Tg(Thy1-CFP/COX8A)S2Lich/J) by two subcutaneous injections, 6 days apart, of an emulsion prepared from saline solutions of peripheral bovine myelin (2.5 mg/mouse), P0_106–125_ (50 μg/mouse) (Genosphere Biotechnologies, Paris, France) and P2_51–84_ (50 μg/mouse) (a kind gift from Dr. Norman A Gregson) and incomplete Freund’s adjuvant (Sigma, Poole, UK) supplemented with 0.5 mg of heat-inactivated *Mycobacterium tuberculosis* (Difco, USA). The injections were carried out under isoflurane anesthesia (2% in oxygen). In addition, 200 ng of pertussis toxin (Chemicon, UK) was administered intraperitoneally to each mouse, both at the same time as the first immunization and also 1 day later. At the same time, control mice received the same amount of emulsion prepared from saline and the same doses of adjuvants. Animals were daily weighed and assessed for disease severity using a 10-point scale, one point being given for each of the following signs: reduction of muscle tone of tail tip, reduction of muscle tone of the whole tail, tail paralysis, inability to spread toes, unsteady gait, hind limb paresis (one point per limb), limb paralysis (one point per limb), and moribund. The cumulative severity of motor deficit that each animal exhibited was expressed as the area under the curve of that animal’s motor deficit against time, as calculated using GraphPad Prism software.

### Surgical procedure and imaging

On the first day of appearance of neurological signs of EAN (symptomatic EAN group), typically week tip of the tail, mice were anesthetized (no recovery; urethane; 1.25 g/kg) for imaging studies, as previously described [26]. On the same day, or a day after, an adjuvant-only control mouse was examined in the same way. Briefly, the skin was opened under urethane anesthesia at the thigh and the connective tissue carefully removed. Approximately 1 cm of the left saphenous nerve was exposed in the middle of the thigh, and desheathed taking care to avoid any damage to the nerve fibers or the vasculature. The nerve was incubated in situ with tetramethylrhodamine methyl ester (TMRM; 0.5 μM solution in sterile saline; pH = 7.4) for 45 min, followed by Griffonia IB4 (Sigma, UK, 1:20 in sterile saline) for 10 min. Labelling with TMRM in these mice enabled distinguishing between two populations of mitochondria, polarized (healthy) and depolarized (damaged). Thus, as a potentiometric, kationic dye, TMRM accumulates only in mitochondria with preserved membrane potential. Thus, TMRM^+^ CFP^+^ mitochondria are considered healthy and in confocal images appear pale pink or white. In contrast, CFP is constitutively expressed in all axonal mitochondria regardless of their membrane potential; thus, TMRM^−^, CFP^+^ mitochondria are depolarized and are considered damaged (in confocal images, they appear cyan). Following the labelling, the animal was transferred to the customized stage of a confocal microscope (LSM Pascal 5.0, Zeiss, Germany). Time-lapse or single images of the middle portion of the exposed nerve in the thigh were acquired at 1.97 s per frame using LSM Pascal 5.0 software (Zeiss, Germany) and Zeiss Apochromat Plan × 63 (oil) warmed (36 °C) objective (NA 1.4). The rectal temperature was continuously monitored and maintained at 36 °C by an underlying heating mat. In total, a minimum of three animals from each group were examined.

### Focal, photo-toxic damage of axonal mitochondria in vivo

An additional three asymptomatic EAN animals were used to study the effects of photo-toxic damage to axonal mitochondria, in vivo. The saphenous nerve was surgically prepared and labeled with TMRM and IB4, as described above. Three adjacent regions, each measuring 124 μm^2^ (corresponding the field of view under × 63 objective), were identified along the saphenous nerve (Fig. 3). In order to determine the baseline rate of mitochondrial transport within those regions, time-lapse images of axonal mitochondria were taken as described above. Next, the central of the three regions was photo-bleached by repeated scanning using laser light at 561 nm. The process was repeated until TMRM was entirely photo-bleached. After photo-bleaching, time-lapse images were taken again in the same three regions, i.e., the central, and proximal and distal to central, in order to evaluate mitochondrial function and transport in the same axons as before the photo-toxic damage.

### Image analysis

The number, direction, velocity of moving, and the size of axonal mitochondria in the time-lapse videos were analyzed by a blinded examiner using ImageJ. As CFP is present in both healthy and damaged mitochondria, these measurements were performed on the red channel (i.e., the TMRM labelling) to exclude damaged mitochondria from skewing the analyses of mitochondrial transport. However, the CFP labelling was used to confirm that the analyzed particles were indeed axonal, and not Schwann cell, mitochondria. Also, mitochondrial length in axons was determined in both channels, separately. Internodal segments of myelinated axons with caliber between 4 and 7 μm were first straightened using the Straighten plug-in (http://rsbweb.nih.gov/ij/plugins/straighten.html) for ImageJ version 1.43 software (NIH, Bethesda, MD, USA, rsb.info.nih.gov/ij/). Straightened axons were processed using the Difference Tracker plug-in (https://www.bioinformatics.babraham.ac.uk/projects/difference_tracker/) for ImageJ as previously described [50, 51], and fluorescent particles with a lower cutoff of 5 pixels moving for at least six consecutive frames were considered to be moving mitochondria. The lower cutoff was determined by examination of the size of the smallest moving CFP and TMRM^+^ particles. The number of moving mitochondria and their direction and velocity were directly derived from the results table generated by the Mass Particle Tracker component of the Difference Tracker plug-in. Length of stationary mitochondria was analyzed using the ImageJ Analyse Particle plug-in. First, time-lapse images of axons were straightened using the Straighten plug-in, the background was reduced using intensity subtraction (the radius of the rolling ball was set to 5) in ImageJ, and the time lapse was converted into a binary form. Using the drawing tool, all the moving particles were eliminated. The dimensions of the remaining stationary particles were analyzed using the Analyse Particle plug-in for ImageJ, whereby the Feret’s diameter value was converted into length in micrometers.

### Data analysis

Prior to all statistical analyses, the data were tested for normality of distribution using the D’Agostino and Pearson normality test. To calculate differences between the number of track counts and the velocity of mitochondrial transport in different groups of animals, non-parametric one-way ANOVA with Dunn’s multiple comparison post-test was used. However, to calculate the difference between the number of mobile mitochondria before and after laser photo-toxic damage, we used repeated measures ANOVA. Differences in length of mitochondria are tense change calculated using one-way ANOVA with Dunn’s multiple comparison tests. Differences between the numbers of small-diameter axons with detected accumulations in different groups were calculated using Student’s *t* test. Parametric data are presented as mean ± SEM, whereas non-parametric, or data which did not follow a normal distribution, are presented as median ± interquartile range (IQR).

## Additional files

Additional file 1: **Video S1.** High-power time-lapse video of inflamed saphenous nerve on the first day of onset of EAN taken immediately upon exposing the saphenous nerve. Axonal myelin (clearly visible around node of Ranvier) is labeled with IB4^+^ (green) and functional mitochondria are labeled with TMRM (red). Some of the axonal mitochondria are mobile. In between axons, a number of infiltrating inflammatory cells labeled with IB4^+^ (surface label, green arrows) can be seen, including the mitochondria (TMRM, red) within the cells. Most inflammatory cells appear freely mobile and moving in typical amoeboid fashion between the axons. In one axon (white arrowhead), functional mitochondria appear focally accumulated, as opposed to uniformly distributed mitochondrial in surrounding axons. One of the inflammatory cells, encircled, can be seen juxtaposed to this focal accumulation. (AVI 3211 kb)

Additional file 2: **Video S2.** High-power time-lapse video of inflamed saphenous nerve on the first day of onset of EAN, taken 40 min after the exposure of saphenous nerve. Forty minutes following the first time-lapse video (Additional file 1: Video S1), the same axons are apparent in the field of view. Some mobile inflammatory cells (surface labeled with IB4^+^, green) and functional mitochondria (TMRM, red) can be seen. Notably, one inflammatory cell (encircled) can be seen closely juxtaposed to the same axon as seen 40 min earlier, still stationary. This is likely to be the same cell, remaining in the close proximity to the axon throughout the 40-min period. The accumulation of axonal mitochondria within this axon appear extended, with some loss of bright red TMRM labelling (mitochondrial membrane potential), suggesting possible mitochondrial damage in that area. (AVI 2879 kb)

Additional file 3: **Video S3.** High-power time-lapse video of axonal mitochondria labeled with TMRM on the day of onset of EAN taken immediately upon exposing the saphenous nerve. Focal accumulation of functional axonal mitochondria (yellow arrow) is observed, with mitochondria moving in anterograde direction. Distal to the accumulation, axons appear depleted of functional mitochondria. In contrast, mitochondrial distribution and morphology appears normal in another axon (green arrow) which is of larger caliber than the two axons affected by mitochondrial accumulation. The video is shown in gray scale in order to improve contrast. (AVI 3253 kb)

Additional file 4: **Video S4.** High-power time-lapse video of axonal mitochondria labeled with TMRM on the day of onset of EAN taken 80 min upon exposing the saphenous nerve. Eighty minutes following the Additional file 3: Video S3, the focal accumulations of functional axonal mitochondria (yellow arrow) appear enlarged in comparison with the earlier time point. Anterograde mitochondrial movement in these axons seems unaffected. Notably, in an axon positioned between the two axons with accumulations (green arrow) and which is of larger diameter than the two indicated with yellow arrows, mitochondrial distribution and morphology appear normal. The video is shown in gray scale in order to improve contrast. (AVI 1353 kb)

Additional file 5: **Video S5.** High-power time-lapse video of axonal mitochondria labeled with TMRM immediately following laser-induced phototoxic damage to mitochondria. The side of the imaging field left to the white line was not exposed to photo-toxic damage. The imaging field to the right of the white line was exposed to laser-induced photo-toxic damage, using the red laser to specifically damage functional mitochondria. Notably, the number of mobile mitochondria transported into the area exposed to photo-toxic damage appears overwhelmingly biased in the favor of anterograde movement. (AVI 2466 kb)

## Abbreviations

ADP: Adenosine diphosphate
ANOVA: Analysis of variance
ATP: Adenosine triphosphate
CFP: Cyan fluorescent protein
CNS: Central nervous system
DRG: Dorsal root ganglion
EAE: Experimental autoimmune encephalomyelitis
EAN: Experimental autoimmune neuritis
GBS: Guillain-Barré syndrome
HIV: Human immunodeficiency virus
IB4: Isolectin B4
IENF: Intra-epidermal nerve fibers
IQR: Interquartile range
LPS: Lipopolysaccharide
LSM: Laser scanning microscope
NA: Numerical aperture
PINK1: PTEN-induced putative kinase 1
PNS: Peripheral nervous system
SD: Standard deviation
SEM: Standard error of the mean
SFN: Small fiber neuropathy
TMRM: Tetramethylrhodamine methyl ester
